# Accumulation of Carotenoids and Metabolic Profiling in Different Cultivars of *Tagetes* Flowers

**DOI:** 10.3390/molecules22020313

**Published:** 2017-02-18

**Authors:** Yun Ji Park, Soo-Yun Park, Mariadhas Valan Arasu, Naif Abdullah Al-Dhabi, Hyung-geun Ahn, Jae Kwang Kim, Sang Un Park

**Affiliations:** 1 Department of Crop Science, Chungnam National University, 99 Daehak-ro, Yuseong-gu, Daejeon 34134, Korea; yunji0825@hanmail.net; 2 National Institute of Agricultural Science, Rural Development Administration, Wanju-gun, Jeollabuk-do 565-851, Korea; psy22@korea.kr; 3 Department of Botany and Microbiology, Addiriyah Chair for Environmental Studies, College of Science, King Saud University, P. O. Box 2455, Riyadh 11451, Saudi Arabia; mvalanarasu@gmail.com (M.V.A.); naldhabi@ksu.edu.sa (N.A.A.-D.); 4 Science & Technology Policy Division, Ministry of Agriculture, Food, and Rural Affairs, Sejong-si 30110, Korea; hgahn@korea.kr; 5 Division of Life Sciences and Convergence Research Center for Insect Vectors, Incheon National University, Incheon 406-772, Korea

**Keywords:** *Tagetes*, marigold, carotenoid, metabolic profiling, GC-TOFMS

## Abstract

Species of *Tagetes*, which belong to the family Asteraceae show different characteristics including, bloom size, shape, and color; plant size; and leaf shape. In this study, we determined the differences in primary metabolites and carotenoid yields among six cultivars from two *Tagetes* species, *T. erecta* and *T. patula*. In total, we detected seven carotenoids in the examined cultivars: violaxanthin, lutein, zeaxanthin, α-carotene, β-carotene, 9-*cis*-β-carotene, and 13-*cis*-β-carotene. In all the cultivars, lutein was the most abundant carotenoid. Furthermore, the contents of each carotenoid in flowers varied depending on the cultivar. Principal component analysis (PCA) facilitated metabolic discrimination between *Tagetes* cultivars, with the exception of Inca Yellow and Discovery Orange. Moreover, PCA and orthogonal projection to latent structure-discriminant analysis (OPLS-DA) results provided a clear discrimination between *T. erecta* and *T. patula*. Primary metabolites, including xylose, citric acid, valine, glycine, and galactose were the main components facilitating separation of the species. Positive relationships were apparent between carbon-rich metabolites, including those of the TCA cycle and sugar metabolism, and carotenoids.

## 1. Introduction

**Figure 1:**
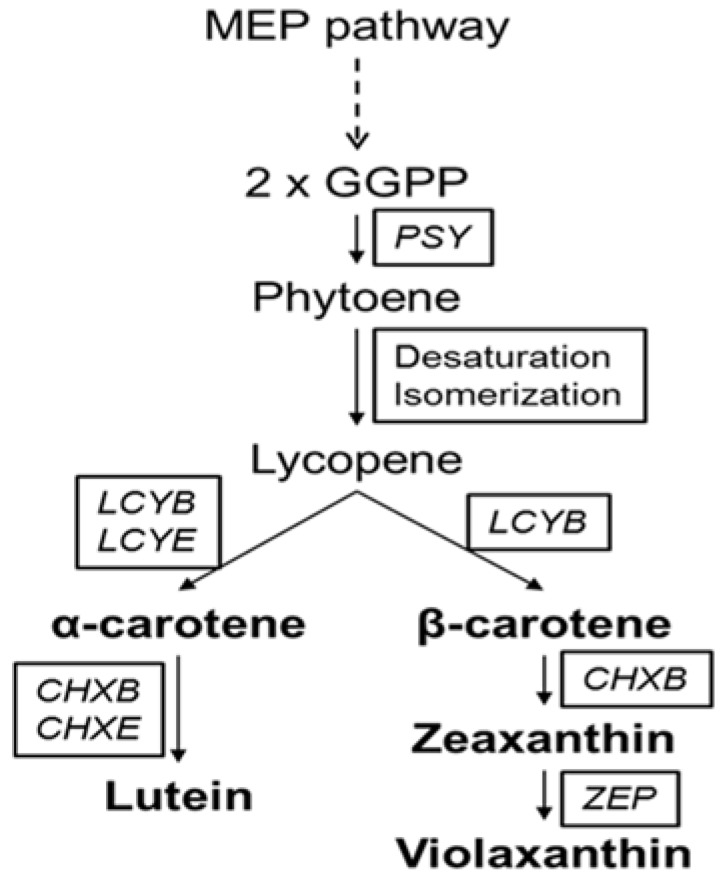
Biosynthetic pathway of the major carotenoids in *Tagetes* cultivars. CHXB, β-ring hydroxylase; CHXE, α-ring hydroxylase; GGPP, geranylgeranyl diphosphate; LCYB, lycopene β-cyclase; LCYE, lycopene ε-cyclase; MEP, methylerythritol; PSY, phytoene synthase; ZEP, zeaxanthin epoxidase.

Carotenoids are natural pigments that contribute to the characteristic yellow, orange, and reddish colors of plant tissues including leaves, fruits, vegetables, and flowers [1]. They play crucial roles in photosynthesis, photoprotection, development, as stress hormones, and signaling molecules in plants [2]. In addition, these colors serve to attract pollinating and seed dispersal agents [3]. Several carotenoids act as precursors of vitamin A, which is an efficient antioxidant and is important for human nutrition. Owing to this property, consumption of carotenoid-rich food is considered to offer protection against some cancers, UV-induced skin damage, coronary heart disease, cataracts, and molecular degeneration [4]. Furthermore, carotenoids are commercially utilized in the agricultural, food, pharmaceutical, and cosmetic industries [5]. The carotenoid biosynthetic pathway has been well characterized on the basis of previous reports on various organisms [6]. In plants, carotenoids are synthesized by nuclear-encoded enzymes in plastid membranes [7]. The pathway begins with condensation of two molecules of geranylgeranyl diphosphate (GGPP), derived from the upstream methylerythritol (MEP) pathway, to form the C_40_ phytoene (Figure 1), in a step mediated by phytoene synthase (*PSY*) [2]. Phytoene is then converted into lycopene by continuous modification, which includes poly-*cis* desaturation and isomerization [8]. Subsequently, cyclization of lycopene leads to the synthesis of various carotenes [5]. α-Carotene is produced from lycopene via the combined action of lycopene β-cyclase (*LCYB*) and lycopene ε-cyclase (*LCYE*). Lycopene can also be converted to β-carotene by *LCYB*. α-Carotene and β-carotene are hydroxylated to lutein and zeaxanthin by β-ring hydroxylase (*CHXB*) and α-ring hydroxylase (*CHXE*), respectively. Violaxanthin is catalyzed by zeaxanthin epoxidase (*ZEP*). Most of the β-carotene in Nature occurs as the *trans* form, although *cis* form β-carotene also occurs in food [9].

Various complex biosynthetic and catabolic pathways are involved in the synthesis of end products and by-products that are detected as metabolites in living organisms [10]. Currently, metabolic profiling has become a valuable tool for the study of changes in metabolites in response to specific treatments or states in biological systems [11,12]. It has been widely utilized in numerous areas of plant research including plant-derived food analysis [13], crop metabolite profiling [14], plant metabolite analysis [15,16], and the development of plant-derived medicines [17]. There are several benefits to be gained from the metabolic profiling of plants. For instance, we expect to gain more information on the biosynthesis of plant natural products, improvements in fields such as plant toxicology and allelopathy, and reductions in the cost of metabolite analysis [12]. The chemical space related to endogenous metabolites, which consists of organic acids, amino acids, amines, sugars, steroids, nucleic acid bases, and other substances is extremely large and diversified [10]. Essentially, three types of technologies such as photo detection, nuclear magnetic resonance (NMR), and mass spectrometry (MS) can be distinguished depending on the detector used [18]. Both the NMR and MS methods provide extensive structural and conformational data for comprehensive metabolic profiling [19]. A variety of techniques, including gas chromatography-mass spectrometry (GC-MS), gas chromatography-time-of-flight mass spectrometry (GC-TOFMS), and liquid chromatography-mass spectrometry (LC-MS), have gained wide acceptance as standard methods for metabolite analysis. Of these, GC-TOFMS has many advantages, including rapid scan times, high peak deconvolution (which is the capability to resolve overlapping peaks), and high sample throughput [20].

Species of *Tagetes* (Asteraceae), which are commonly known as marigolds, are grown for medicinal and ornamental purposes around the world. In addition, the nematocidal, fungicidal, and insecticidal properties of extracts from these species have been demonstrated in several studies [21]. Extracts derived from *Tagetes* species have been shown to exert diverse pharmacological actions, including anti-bacterial, antimicrobial, anti-oxidant, hepatoprotective, wound healing, and larvicidal activities. Some of the major species of *Tagetes*, include *T. erecta* (African or American marigolds), *T. patula* (French marigolds), and *T. signata* ‘pumila’ (Signet marigolds). Each species of *Tagetes* can be distinguished on the basis of different features such as bloom size, shape, and color; plant size; and leaf shape [22]. Furthermore, numerous phytochemicals have been identified in these species, including terpenoids, flavonoids, alkaloids, polyacetylenes, and fatty acids [23]. Of these, carotenoids and flavonoids which are components of *Tagetes* pigments, are the major bioactive constituents of interest [21]. Different tissues of these species have been used in folk medicine for various diseases, including colic diarrhea, vomiting, fever, skin disease, and hepatic disorders [24,25]. In particular, flowers are effective parts that can be used as an astringent, carminative, or stomachic, and for the treatment of fevers, epileptic fits (Ayurveda), scabies, liver complaints, and diseases of the eye [26].

**Figure 2:**
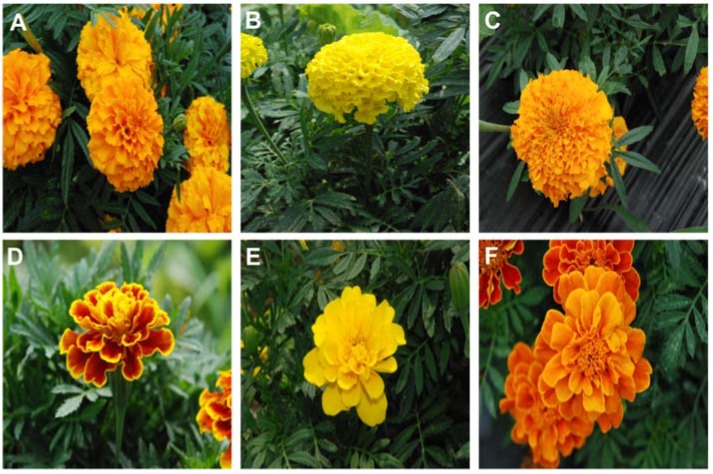
Flowers of different *Tagetes* cultivars grown in Chungnam National University, South Korea. Photographs were taken by Y.J. Park. (**A**): Discovery Orange; (**B**): Inca Yellow; (**C**): Inca Orange; (**D**): Durango Bee; (**E**): Durango Yellow; (**F**): Safari Red.

The goal of the present study was to investigate the differences in metabolite composition amongst six cultivars of marigold, *T. erecta* ‘Discovery Orange’, ‘Inca Orange’, and ‘Inca Yellow’; and *T. patula* ‘Durango Bee’, ‘Durango Yellow’, and ‘Safari Red’ (Figure 2). Carotenoids were identified and quantified by high-performance liquid chromatography (HPLC). In addition, we performed metabolic profiling using GC-TOFMS.

## 2. Results and Discussion

### 2.1. Analysis of Carotenoid Content 

**Figure 3:**
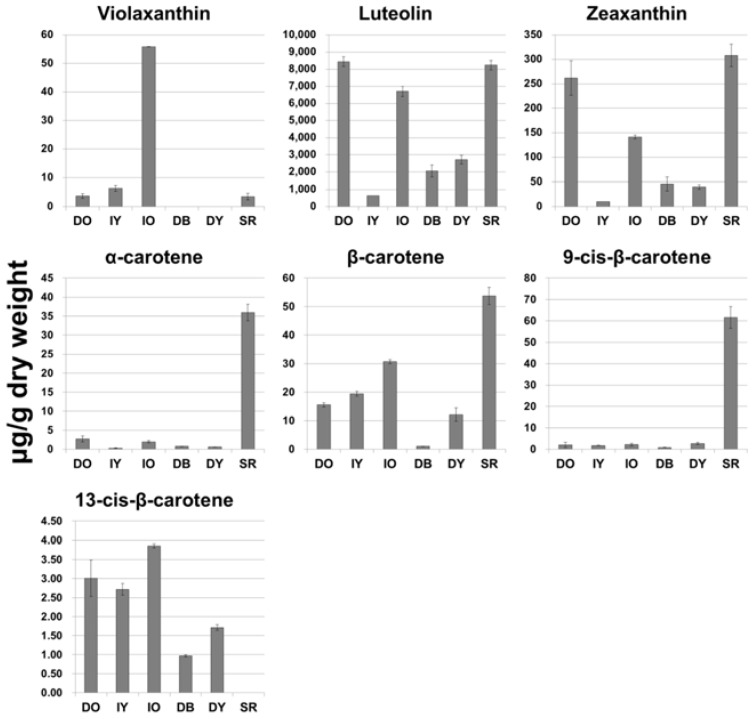
Quantitative results (μg/g dry weight) for carotenoids in the flowers of different *Tagetes* cultivars. DO, Discovery Orange; IY, Inca Yellow; IO, Inca Orange; DB, Durango Bee; DY, Durango Yellow; SR, Safari Red.

The color of plants tissues can vary from yellow to red to orange, depending on the number of conjugated double bonds and the diverse functional groups in carotenoid molecules [27,28,29]. Carotenes, which are simple carotenoid forms, contain various isomeric structures, including α, β, γ, δ, ε, and ζ [30]. Of these, α- and β-carotenes are the primary forms of carotenes. Xanthophylls, which are the oxidized derivatives of carotenes, are mostly found in the leaf, and impart yellow to red colors [31]. Lutein, zeaxanthin, and cryptoxanthin are the commonly occurring xanthophylls [32] with fruits and vegetables being particularly abundant in lutein [33,34]. In the present study, we monitored the carotenoids in flower extracts from six cultivars of *Tagetes* using HPLC analysis. In total, seven carotenoids (four types of carotenes: α-carotene, β-carotene, 9-*cis*-β-carotene, and 13-*cis*-β-carotene; and three types of xanthophylls: violaxanthin, lutein, and zeaxanthin) were detected in *Tagetes* (Figure 3). 

We detected considerable differences in carotenoid composition, depending on cultivar and type of carotenoid. The highest concentration of violaxanthin, which imparts an orange color in plants, was detected in Inca Orange (IO), whereas this compound was not detected in Durango Bee (DB) or Durango Yellow (DY). Cultivars with yellow-colored flowers, such as Inca Yellow (IY), DB, and DY, exhibited low levels of lutein. However, those with orange-colored flowers, Discovery Orange (DO), IO, and Safari Red (SR) showed high levels of lutein. Furthermore, a similar pattern was found with respect to zeaxanthin measurements. α-Carotene was significantly accumulated in SR compared to other cultivars. The highest amount of β-carotene was also found in SR, followed by IO, IY, DO, DY, and DB. Similarly, the highest and lowest amounts of 9-*cis*-β-carotene were detected in SR and DB, respectively. Interestingly, in all cultivars, 13-*cis*-β-carotene showed the same pattern as β-carotene; the exception being SR, in which 13-*cis*-β-carotene was not detected. We found that lutein is the main pigment in *Tagetes* flower extracts. Similarly, a previous study has demonstrated that *T. erecta* contains lutein and zeaxanthin as the major carotenoids, which represent more than 10% of the total carotenoids [35]. Furthermore, early work indicated that galenine, lutein, lycopene, α-carotene, β-carotene, and γ-carotene comprise 6% of the carotenoids in dried *T. patula* flowers [36]. 

There are various factors that have an effect on the qualitative and quantitative differences in carotenoid contents, including cultivar/variety, developmental stage, climate or geographic features of the production area, agricultural conditions, and post-harvest handling, processing, and storage conditions [29]. Many studies have investigated carotenoid biosynthesis during different developmental stages and/or amongst cultivars showing different colors [5]. In this regard, compositional differences among cultivars have been particularly well demonstrated in recent years. For example, the carotenoid content of marigold cultivars was shown to differ by more than 100-fold [37]. Similarly, previous studies have determined that different cultivars of vegetables such as sweet potato, squash, and pumpkin produce variable concentrations of carotenoids [29].

### 2.2. Chromatographic Data from GC-TOFMS

**Figure 4:**
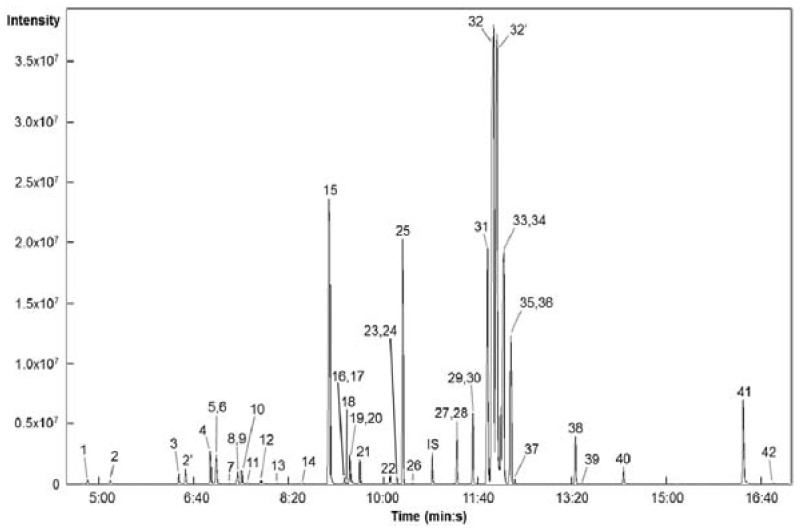
Selected ion chromatograms of metabolites extracted from *Tagetes* species. 1, lactic acid; 2, valine; 3, glycolic acid; 4, serine; 5, ethanolamine; 6, glycerol; 7, isoleucine; 8, nicotinic acid; 9, glycine; 10, succinic acid; 11, glyceric acid; 12, fumaric acid; 13, threonine; 14, β-alanine; 15, malic acid; 16, salicylic acid; 17, aspartic acid; 18, methionine; 19, pyroglutamic acid; 20, 4-aminobutyric acid; 21, threonic acid; 22, glutamic acid; 23, phenylalanine; 24, p-hydroxybenzoic acid; 25, xylose; 26, asparagine; 27, vanillic acid; 28, glutamine; 29, shikimic acid; 30, citric acid; 31, quinic acid; 32, fructose; 33, galactose; 34, glucose; 35, syringic acid; 36, mannose; 37, mannitol; 38, inositol; 39, ferulic acid; 40, tryptophan; 41, sucrose; 42, trehalose; IS, internal standard (ribitol).

Metabolic profiling provides a global overview of plant metabolic phenotypes [15,16]. In particular, GC-MS has been increasingly applied in metabolomics studies because the capillary columns used in GC enable the separation of more than 100 compounds in a single analysis [38]. Therefore, we used GC-TOFMS to identify and quantify the low-molecular-weight hydrophilic compounds present in *Tagetes* flowers. In terms of GC-MS data, deconvolution is the process of computationally separating co-eluting components and creating a pure spectrum for each component. Commercial software tools, such as ChromaTOF and AMDIS have been developed for this purpose [38]. In this study, we used ChromaTOF software for peak deconvolution. Peak identification was performed by matching the *m*/*z* and retention time values of reference compounds (Figure 4). In total, 42 metabolites-four phenolics (ferulic, *p*-hydroxybenzoic, syringic, and vanillic acids) and 38 primary metabolites-were detected in marigolds. The corresponding retention times are illustrated in Table S1 and are consistent with our previous data [39].

### 2.3. PCA and OPLS-DA

**Figure 5:**
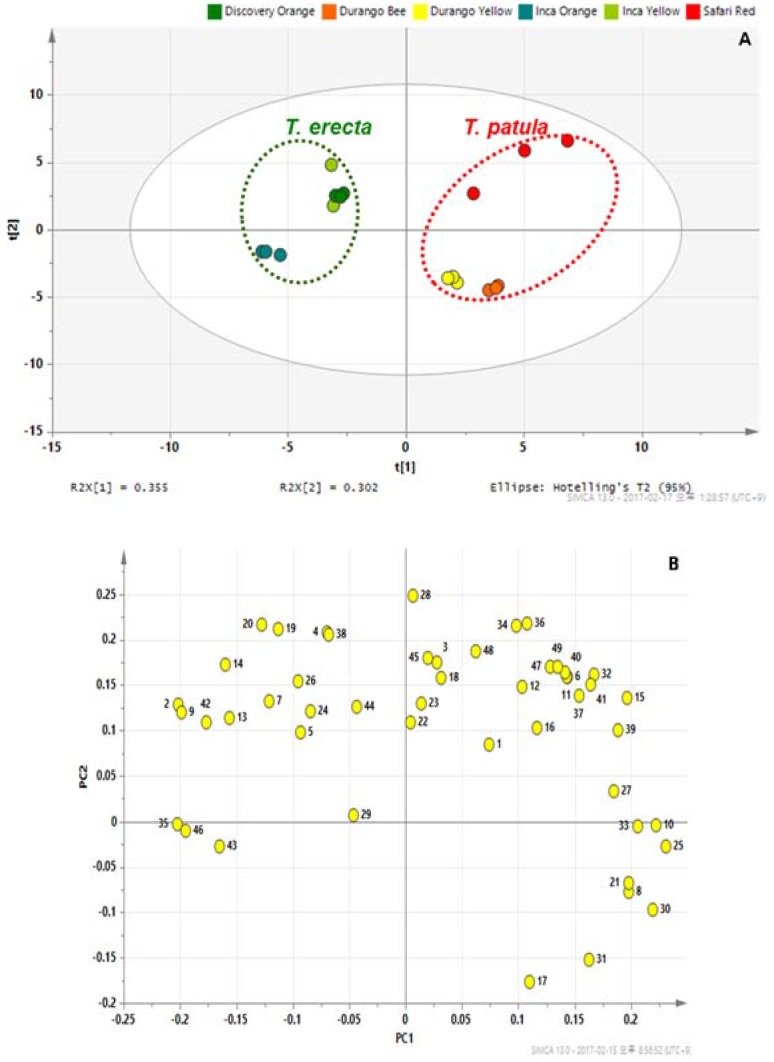
Results of a PCA of the metabolite data. (**A**) Score plot; (**B**) Loading plot. PC1, prinicipal component 1; PC2, prinicipal component 2. 1, Lactic acid; 2, Valine; 3, Glycolic acid; 4, Serine; 5, Ethanolamine; 6, Glycerol; 7, Isoleucine; 8, Nicotinic acid; 9, Glycine; 10, Succinic acid; 11, Glyceric acid; 12, Fumaric acid; 13, Threonine; 14, β-Alanine; 15, Malic acid; 16, Salicylic acid; 17, Aspartic acid; 18, Methionine; 19, Pyroglutamic acid; 20, 4-Aminobutyric acid; 21, Threonic acid; 22, Glutamic acid; 23, Phenylalanine; 24, p-Hydroxybenzoic acid; 25, Xylose; 26, Asparagine; 27, Vanillic acid; 28, Glutamine; 29, Shikimic acid; 30, Citric acid; 31, Quinic acid; 32, Fructose; 33, Galactose; 34, Glucose; 35, Syringic acid; 36, Mannose; 37, Mannitol; 38, Inositol; 39, Ferulic acid; 40, Tryptophan; 41, Sucrose; 42, Trehalose; 43, Violaxanthin; 44, Lutein; 45, Zeaxanthin; 46, 13-*cis*-β-Carotene; 47, α-Carotene; 48, β-Carotene; 49, 9-*cis*-β-Carotene.

Metabolomics allows the rapid assessment of metabolites and the identification of biomarkers for discrimination of samples based on the genotype and environmental conditions [40]. Quantification data of the 42 identified metabolites, which were normalized based on IS signal intensity, and seven carotenoids were subjected to PCA to identify differences in metabolite profiles among the examined cultivars (Figure 5). The PCA was performed on the 49 metabolites with standardization pre-processing. Core primary metabolites offer a great opportunity to discriminate metabolites between different genotypes [41]. The quality of the PCA models is described by R^2^ values [42], which are defined as the proportions of variance in the data explained by the model and represent the goodness of fit. R^2^ values of 0.657 were investigated from the PCA model using two components. The Q^2^ value for the PCA model was 0.439. With the exception of cultivars IY and DO, separation between cultivars was apparently performed by PCA. Furthermore, principal component 1 (PC1) resolved *T. erecta* from *T. patula*. 

Thus, the effects of cultivars primarily contribute to the total variance within the data set. The loading plots highlighted and visualized metabolites with a significant role in cultivar separation. To further investigate the contributors to the principal components, the metabolic loadings in PC1 were xylose, succinic acid, citric acid, and galactose, for which the eigenvectors were 0.23105, 0.22183, 0.2195, and 0.20624, respectively. Besides, in PC1, the corresponding loading was positive for all sugars, with the exception of trehalose.

**Figure 6:**
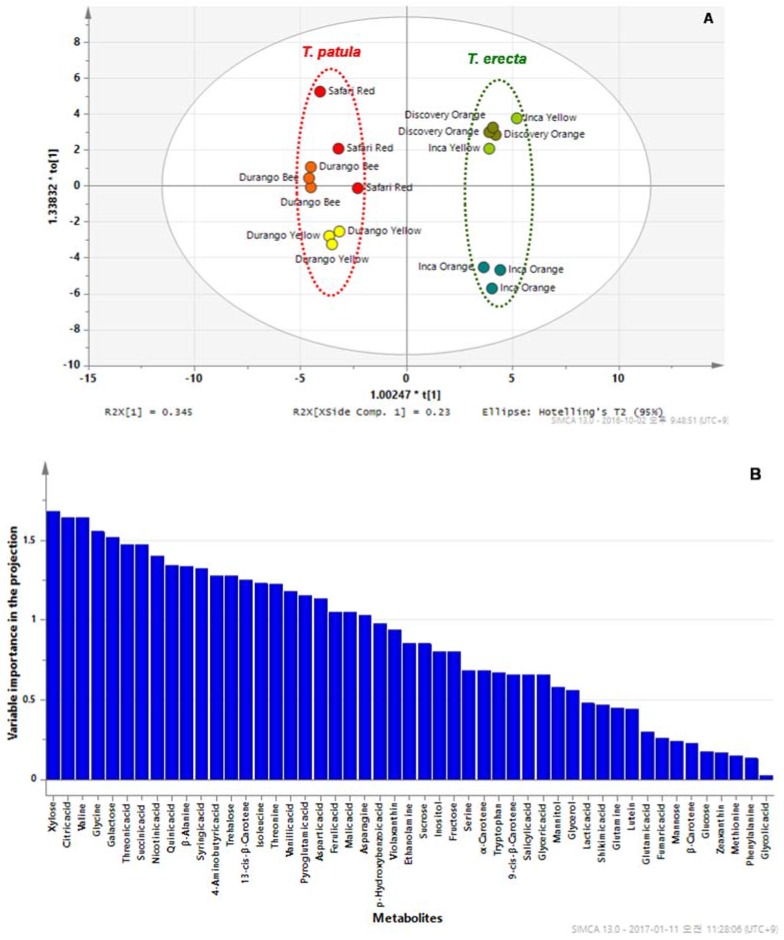
Results of an OPLS-DA of the metabolite data. (**A**) Score plot; (**B**) Variable importance in the projection (VIP).

In principal component 2 (PC2), glutamine had a strong impact on the separation. PCA showed that, with the exception of trehalose, all sugars and most carotenoids were clustered on the right side of the loading plot. We found that sugars as well as zeaxanthin and β-carotene were higher in SR than in the other cultivars. To maximize the separation between *T. erecta* and *T. patula*, OPLS-DA was applied (Figure 6). 

**Figure 7:**
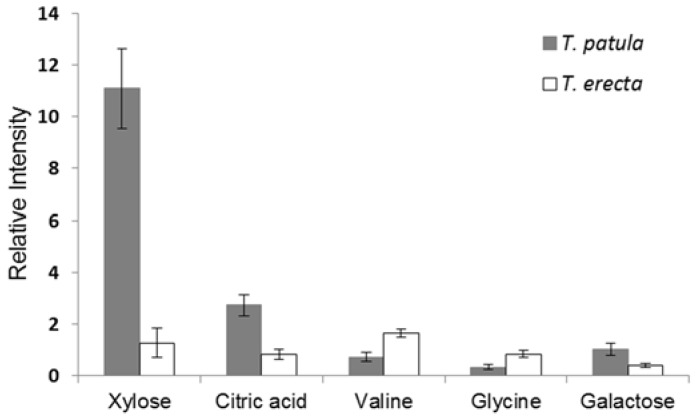
Relative quantification of metabolites that were significantly different (*p* < 0.0001) between *T. patula* and *T. erecta*. Data are represented as the mean ± SD.

The model was constructed with a Q^2^ (cross-validated predictive ability [42]) value of 0.956, indicating that the model is considered to have an excellent predictive ability since Q^2^ > 0.9. In addition, we performed an external validation to test the validity of the OPLS-DA model. For the prediction, the four samples (a test data set) were left and the OPLS-DA was established with the training samples. The R^2^Y_cum_ and Q^2^Y_cum_ value was 1.0 and 0.918, respectively. Variable importance in the projection (VIP) is a weighted sum of squares of the OPLS weight [42]. As seen in the results, the significantly distinguishing metabolites are summarized according to VIP > 1.5. Xylose, citric acid, valine, glycine, and galactose were found to differentiate significantly (*p* < 0.0001) between the two species (Figure 7). Thus, we found that core primary metabolites could provide useful information (biomarker) for the discrimination of *Tagetes* species. 

### 2.4. Hierarchical clustering analysis (HCA)

**Figure 8:**
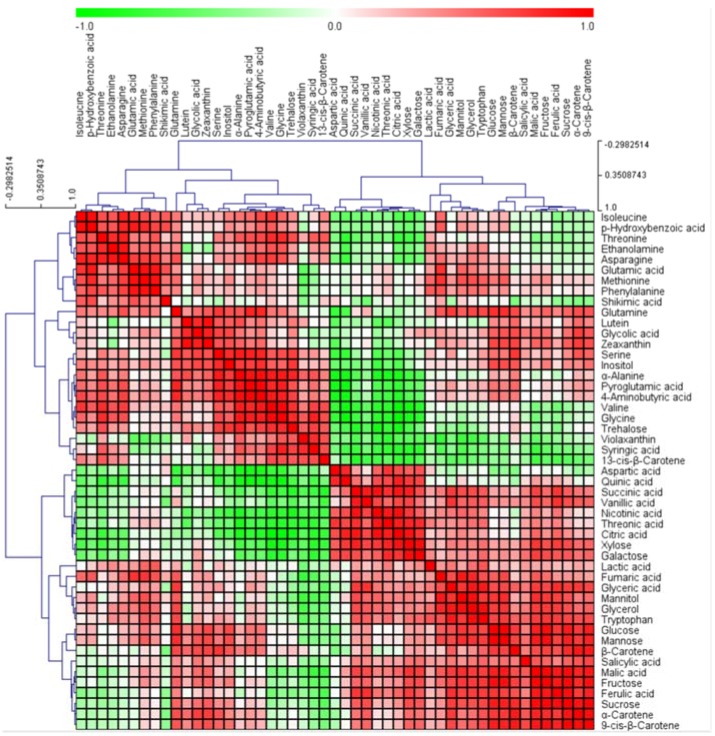
Correlation matrix of 49 metabolites from six cultivars of marigold. Each square indicates *r* (Pearson’s correlation coefficient values of a pair of compounds). On the basis of the intensity of red and green colors, the value of correlation coefficient is reflected. Hierarchical clusters are represented by a cluster tree.

Significant correlation between metabolites may represent an extensive coordination among biosynthetic pathways [43]. Pearson correlation analysis was used to analyze the metabolite-metabolite correlation among low-molecular-weight metabolites in *Tagetes* flowers. An HCA of these correlation coefficients was conducted to visualize the detailed relationships between metabolites (Figure 8). 

The correlation data revealed some interesting correlative metabolic networks for biochemical reactions in plants. These metabolites grouped into two correlation clusters. Cluster 1 contained all the amino acids apart from aspartic acid, whereas Cluster 2 contained most carbon-rich metabolites related to the TCA cycle and sugar metabolism. Carbon and nitrogen metabolism are closely interlinked [44], and changes in the sugar supply lead to coordinated reprogramming of carbon and nitrogen metabolism with sugar depletion leading to an inhibition of nitrate assimilation [44]. A previous study found that an increase in the carbon:nitrogen ratio under conditions of elevated carbon dioxide is mainly attributable to a decrease in nitrate [45]. Furthermore, there is increasing evidence that the MEP pathway can be regulated by components of the nitrate assimilation pathway [46]. Carotenoids, which are carbon-rich products, are not only end products of the chloroplast assimilation of photosynthetic carbon but also by virtue of the phytyl side chain of chlorophyll. In this study, we found that most carotenoids were positively correlated with intermediates of the TCA cycle and sugars. The HCA results provided correlations between the metabolites involved in closely related pathways. Carotenoids are essential accessory light-harvesting pigments. Plants synthesize carbohydrates from carbon dioxide and water by photosynthesis, which C metabolism (e.g., sugar metabolism, glycolysis, and TCA cycle) is responsible for the production of accessible energy. When the carbon or nitrogen supply is changed, many central metabolites in carbon and nitrogen metabolism change in parallel rather than antagonistically [44], and we similarly found a positive correlation between the levels of amino acids, including gamma-amino butyric acid (GABA) and glutamine, and carotenoids. In our previous study, we found that riper fruits of bitter melon (*Mormordica charantia* Linn) had higher carotenoid concentrations than less ripe fruits [47], and Boggio et al. have similarly reported that the concentrations of glutamate, GABA, and glutamine in tomatoes increase markedly increased during their ripening [48]. Therefore, the present study has clearly demonstrated the potential of metabolomics as a powerful tool for investigating complex metabolic links for systems biology. The main multi-parametric technologies, NMR spectrometry and MS, have been popular, increasingly. NMR spectrometry can give high quantitative and reproducible information, but low-sensitivity. In contrast, MS produces high sensitivity and accurate metabolite identification for detecting metabolites. Recently, the combination of NMR and MS data that leads to great opportunities on advanced identification of unknown analytes and expansive range of metabolomics has been developed [49]. In further study, we will discover the difference and complementary from NMR- and MS-based analyses to improve the comprehensive metabolomics information.

## 3. Materials and Methods

### 3.1. Plant Materials and Sample Preparation

*Tagetes* seeds were obtained from Seed Mall Co. (Seoul, Korea). They were cultivated at the experimental farm of Chungnam National University (Daejeon, Korea) without any regulation to the cultures. Each plant was exposed to outdoor conditions for 6 months. During cultivation, any temperature condition or additional illumination has not been regulated. Flowers of different *Tagetes* cultivars were harvested during the flowering stage in July 2014; in this month the average temperature was 25.9 °C, with a relative humidity of 82.6%, an average precipitation of 177.2 mm, according to the data collected from the Korea Meteorological Administration (http://web.kma.go.kr). Five flowers from the same branch were collected and all samples were prepared as three biological replicates. The separated parts of these plants were flash frozen in liquid nitrogen. The materials were completely freeze-dried at −80 °C for 72 h (Ilshin Lab Co., Ltd., Yangju-si, Korea) and the dried samples were well ground in to milled powder using mortars and pestles.

### 3.2. Carotenoid Extraction and Quantification

Extraction of carotenoids from *Tagetes* species was adapted from Park et al [50]. Solvent (3 mL of ethanol containing 0.1% of ascorbic acid (*w*/*v*)) was added to 0.1 g of sample. The samples were incubated at 85 °C for 5 min. The carotenoid extracts were then saponified with potassium hydroxide (120 μL, 80% *w*/*v*) at 85 °C for 10 min. Saponification lead to removal of fatty acids and residual free pigments, then separation and identification of the carotenoids are possible by HPLC. After saponification, 1.5 mL of deionized water was added to samples in cooling condition. β-apo-8′-carotenal (0.2 mL, 25 μg/mL) was used as an internal standard based on the features and retention time of carotenoid. The samples were separated into two layers using extraction twice with 1.5 mL of hexane at 1200× *g* for 5 min at 4 °C. The combined supernatants were then evaporated under a stream of nitrogen and dissolve in 50:50 (*v*/*v*) dichloromethane/methanol. HPLC analyses were performed on an Agilent 1100 HPLC instrument (Massy, France) coupled to a photodiode array detector. Separation was achieved on a C30 YMC column (250 × 4.6 mm, 3 μm; YMC Co., Kyoto, Japan) and absorption determinations were generated at 450 nm. A methanol/water (92:8 *v*/*v*) containing 10 mM ammonium acetate (solvent A) to 100% methyl *tert*-butyl ether (solvent B) gradient was used at a flow rate of 1 mL/min. The gradient was increased from 10% solvent B to 75% and returned to initial composition. Identification and quantification of carotenoids were determined by comparison with retention time and co-elution with authentic standards. 

### 3.3. Metabolic Profiling

Polar metabolites were extracted from *Tagetes* species by the method presented previously [39]. In order to extract primary metabolites, 1 mL of methanol:water:chloroform (2.5:1:1) was added to 10 mg of the powdered materials. Ribitol (60 μL, 0.2 mg/mL) was added and used as an internal standard (IS). Chemical derivatization, i.e., oximation and trimethylsilyl etherification, was performed on the extracted metabolites in preparation for GC-TOFMS analysis. After derivatization, GC-TOFMS procedures were carried out as described by Kim et al. [39]. Peak detection and automated deconvolution of reference mass spectra were conducted by using ChromaTOF software before quantitative analysis. Compound identification was performed by matching the mass spectra with those in the National Institute of Standards and Technology (NIST) library and in-house libraries for standard chemicals. Quantitative analyses were based on peak area ratio by using the IS ribitol (normalized response).

### 3.4. Statistical Analysis

In order to investigate the relationship between multivariate data groups based on similarity or dissimilarity, we performed PCA and OPLS-DA (SIMCA-P version 13.0; Umetrics, Umeå, Sweden) using the relative quantification data acquired from GC-TOFMS. The PCA output comprised loading plots for the cluster separation and score plots to visualize the differences between samples. The scaled data file with unit variance scaling was subjected to PCA and OPLS-DA for all the variables. Pearson’s correlation analysis was carried out among the relative levels of 49 metabolites with standardization procedures using the SAS 9.2 software package (SAS Institute, Cary, NC, USA). HCA and heat map visualization of the correlation coefficients were performed using the software M_ULTI_E_XPERIMENT_ V_IEWER_ version 4.4.0 (http://www.tm4.org).

## Supplementary Materials

Table S1. Metabolites identified in GC-TOFMS chromatograms of marigold flowers (cv. Durango Yellow) extracts.

## Abbereviations

The following abbreviations are used in this manuscript:

CHXB	β-ring hydroxylase
CHXE	α-ring hydroxylase
DO	Discovery Orange
DB	Durango Bee
DY	Durango Yellow
GABA	γ-Aminobutyric acid
GC-MS	Gas-chromatography-mass-spectrometry
GC-TOFMS	Gas-chromatography-time-of-flight-mass-spectrometry
GGPP	Geranylgeranyldiphosphate
HCA	Hierarchical clustering analysis
HPLC	High-performance liquid chromatography
IO	Inca Orange
IS	Internal standard
IY	Inca Yellow
LC-MS	Liquid-chromatography-mass-spectrometry
LCYB	Lycopene β-clcylase
LCYE	Lycopene ε-cyclase
MEP	Methylerythritol
MS	Mass spectrometry
NIST	National Institute of Standards and Technology
NMR	Nuclear magnetic resonance
OPLS-DA	Orthogonal projection to latent structure-discriminant analysis
PC1	Principal component 1
PC2	Principal component 2
PCA	Principal component analysis
PSY	Phytoene synthase
SR	Safari Red
VIP	Variable importance in the projection
ZEP	Zeaxanthin epoxidase
