# Supplementary material for: Accumulation of Carotenoids and Metabolic Profiling in Different Cultivars of Tagetes Flowers
Source: Molecules. 2017 Feb 18;22(2):313. doi: 10.3390/molecules22020313 (PMC6155894; doi:10.3390/molecules22020313)
Supplement: Supplementary file 1 [file molecules-22-00313-s001.pdf]

Supplementary Materials: Accumulation of Carotenoids and Metabolic Profiling in Different Cultivars of *Tagetes* Flowers

Yun Ji Park [1], Soo-Yun Park [2], MariadhasValanArasu [3], Naif Abdullah Al-Dhabi [3], Hyung-geun Ahn [4], Jae Kwang Kim [5,*], Sang Un Park [1,*]

Table S1: Metabolites identified in GC-TOFMS chromatograms of Marigold flowers (cv. Durango Yellow) extracts

No.[1)]	Compound	RT[2)]	RRT[3)]	Selected ion for quantification[4)]
1	Lactic acid	4.80	0.442	147
2	Valine	5.20	0.479	146
3	Glycolic acid	6.40	0.589	147
4	Serine	6.96	0.641	116
5	Ethanolamine	7.04	0.648	174
6	Glycerol	7.06	0.650	147
7	Isoleucine	7.30	0.672	158
8	Nicotinic acid	7.42	0.683	180
9	Glycine	7.44	0.685	174
10	Succinic acid	7.51	0.692	147
11	Glyceric acid	7.61	0.701	147
12	Fumaric acid	7.85	0.723	245
13	Threonine	8.14	0.750	219
14	*β*-alanine	8.56	0.788	174
15	Malic acid	9.06	0.834	147
16	Salicylic acid	9.31	0.857	267
17	Aspartic acid	9.31	0.857	100
18	Methionine	9.37	0.863	176
19	Pyroglutamic acid	9.41	0.866	156
20	4-aminobutyric acid	9.44	0.869	174
21	Threonic acid	9.59	0.883	147
22	Glutamic acid	10.12	0.932	246
23	Phenylalanine	10.25	0.944	218
24	*p*-Hydroxybenzoic acid	10.27	0.946	223
25	Xylose	10.34	0.952	103
26	Asparagine	10.52	0.969	116
IS	Ribitol	10.86	1.000	217
27	Vanillic acid	11.28	1.039	297
28	Glutamine	11.30	1.041	156
29	Shikimic acid	11.45	1.054	204
30	Citric acid	11.58	1.066	273
31	Quinic acid	11.84	1.090	345
32	Fructose	11.95	1.100	103
33	Galactose	12.07	1.111	147
34	Glucose	12.12	1.116	147

35	Syringic acid	12.22	1.125	297
36	Mannose	12.25	1.128	147
37	Manitol	12.31	1.134	319
38	Inositol	13.39	1.233	305
39	Ferulic acid	13.50	1.243	338
40	Tryptophane	14.23	1.310	202
41	Sucrose	16.34	1.505	217
42	Trehalose	16.88	1.554	191

[1)] Numbers represent the compound index for the chromatogram peaks shown in Figure 4.

[2)] Retention time (min).

[3)] Relative retention time (retention time of the analyte/retention time of the IS).

[4)] Specific mass ion used for quantification.
